# Hybridization of Strength Pareto Multiobjective Optimization with Modified Cuckoo Search Algorithm for Rectangular Array

**DOI:** 10.1038/srep46521

**Published:** 2017-04-20

**Authors:** Khairul Najmy Abdul Rani, Mohamedfareq Abdulmalek, Hasliza A. Rahim, Neoh Siew Chin, Alawiyah Abd Wahab

**Affiliations:** 1 Bioelectromagnetics Research Group (BioEM), Department of Electronics Engineering Technology, Faculty of Engineering Technology, Universiti Malaysia Perlis, Padang Besar, Perlis, Malaysia; 2 Faculty of Engineering and Information Sciences, University of Wollongong in Dubai, Dubai Knowledge Park, Dubai, United Arab Emirates; 3 Bioelectromagnetics Research Group (BioEM), School of Computer and Communications Engineering, Universiti Malaysia Perlis, Pauh Putra, Perlis, Malaysia; 4 Faculty of Engineering, Technology & Built Environment, University College Sedaya International, Kuala Lumpur, Malaysia; 5 School of Computing, Universiti Utara Malaysia, Sintok, Kedah, Malaysia

## Abstract

This research proposes the various versions of modified cuckoo search (MCS) metaheuristic algorithm deploying the strength Pareto evolutionary algorithm (SPEA) multiobjective (MO) optimization technique in rectangular array geometry synthesis. Precisely, the MCS algorithm is proposed by incorporating the Roulette wheel selection operator to choose the initial host nests (individuals) that give better results, adaptive inertia weight to control the positions exploration of the potential best host nests (solutions), and dynamic discovery rate to manage the fraction probability of finding the best host nests in 3-dimensional search space. In addition, the MCS algorithm is hybridized with the particle swarm optimization (PSO) and hill climbing (HC) stochastic techniques along with the standard strength Pareto evolutionary algorithm (SPEA) forming the MCSPSOSPEA and MCSHCSPEA, respectively. All the proposed MCS-based algorithms are examined to perform MO optimization on Zitzler–Deb–Thiele’s (ZDT’s) test functions. Pareto optimum trade-offs are done to generate a set of three non-dominated solutions, which are locations, excitation amplitudes, and excitation phases of array elements, respectively. Overall, simulations demonstrates that the proposed MCSPSOSPEA outperforms other compatible competitors, in gaining a high antenna directivity, small half-power beamwidth (HPBW), low average side lobe level (SLL) suppression, and/or significant predefined nulls mitigation, simultaneously.

Recently, engineers and researchers have done many studies to design and fabricate an array antenna formed by a set of radiating elements or isotropic radiators circumscribed by certain geometrical structure. The antenna array is widely used to detect and process signals arriving from various directions of arrival (DoA) in various fields, such as communications, radar, sonar, and navigation systems due to its higher levels of gain, directivity, and signal-to-noise ratio (SNR) compared to single-antenna structures^1^. Overall, the primary objective of conducting array geometry synthesis is to determine the physical characteristics of the array and selection of elements or radiators, which can emit an electromagnetic radiation pattern almost similar to the desired pattern. The antenna array pattern synthesis problem consists of finding weights that satisfy a set of specifications on the beam pattern^2^. The individual elements of the array can be of any form of wires, and apertures^3^. Then, there will be a determination of the element excitations required for achieving a particular performance, sometimes under a given constraint^4^. Theoretically, no antenna is able to radiate all the energy in one preferred direction. Some energy is inevitably radiate in other directions with lower levels than the main beam. These smaller peaks are referred to as side lobes, commonly are specified in decibel (dB) down from the main lobe. Furthermore, in an antenna radiation pattern, there is a zone in which the effective radiated power is at a minimum known as a null. The null is useful for suppressing interfering signals in a predefined direction^5^. The analysed array pattern ideally should demonstrate a high power gain or directivity, lower side lobe level (SLL), controllable beamwidth^6^, particular pattern characteristics^4^, and good azimuthal symmetry, respectively. This is required to cater the high bandwidth and quality of service (QoS) significant demands for long distance wireless communications^3^. Hence, it is critical to optimize antenna array element locations (based on λ/2 inter-element spacing), excitation current amplitudes, and excitation current phases with a random distribution, respectively^7^.

Evolutionary computing also referred as evolutionary computation (EC) or evolutionary algorithm (EA) is the field of research that draws ideas from evolutionary biology in order to develop search and optimization techniques for solving complex problems^8^. More than 40 years ago, computer scientists and engineers began developing EC/EA to generate solutions to problems, which were too difficult and complicated to tackle using conservative analytical methods. EC/EA then rapidly has become a major field of machine learning and system optimization. EC/EA basically tries to combine basic heuristic methods in exploring optimal solutions in a search space. These heuristic methods are nowadays commonly called “metaheuristics”. The term “metaheuristic” is originally derived from the composition of two Greek words^9^. “Heuristic” comes from the verb “heuriskein”, which means “to find”, while the suffix “meta” means “beyond, in an upper level”. Before this term was widely adopted, metaheuristics were often called modern heuristics^10^. Because of featuring high adaptability, flexibility and capability to optimize complex multidimensional problems with a nonlinear and a nonconvex dependence of design parameters^11^, modern metaheuristic techniques, such as genetic algorithm (GA)^12^, grey wolf optimization (GWO)^13^, artificial neural network (ANN)^14^, and particle swarm optimization (PSO)^15^, have been applied for antenna array synthesis. These EC/EA techniques are proven outperform the original gradient methods and conventional numerical techniques. Moreover, through the stochastic search process, the EC/EA techniques are capable to deal with large number of optimization parameters via relatively easy computer simulations^16^.

## System Description

### Problem Mathematical Formulation for Proposed MCS Algorithms

Recent studies show that cuckoo search (CS) algorithm capable to be an alternative of other existing EC/EA techniques for complex multidimensional optimization due to its high efficiency. An efficient optimization algorithm is critical to ensure that optimal solutions are reachable in search space^17^. For an example, CS algorithm outperforms PSO in achieving the global convergence. In this case, PSO algorithm converges prematurely to a local optimum (stuck in local optima predicament), while CS algorithm converges up to the global optimality for a multimodal optimization^17^. This is because CS algorithm has two crucial search capabilities, which are local search and global search controlled by a fraction probability or discovery rate, *P*_*a*_ internal parameter. Another advantage is CS algorithm uses Lévy flights motion with infinite mean and variance rather than standard random walks motion for global search. Consequently, CS algorithm can explore the search space more efficiently than some EC rivals, which use standard Gaussian process^17^.

As a newly evolved stochastic technique, the CS metaheuristic algorithm is inspired by the obligate brood parasitism behavior of some cuckoo species by laying their eggs in the nests of other host birds (of other species)^18^. Principally, the unique behavior of some host birds reacting to the invading cuckoos becomes the key notion. In this case, if a host bird discovers the eggs are not their own, it will respond either throw these alien eggs away or simply leave its nest, and make a new nest in other place. Conceptually, the CS algorithm acts as a Markov process of animal motion known as Lévy flight^18^, where after a large number of steps, the distance from the origin of random walk tends to reach a stable distribution. Consequently, such behavior has been emulated to perform optimization and global optimal search with a promising capability^19^,^20^.

As a matter of fact, the original CS algorithm has three drawbacks, which have been improved in this study. First, the original CS algorithm uses fixed value for both discovery rate, *P*_*a*_ and Lévy flight Gaussian distribution, *α* parameters. Both internal parameters are critical and sensitive in fine-tuning a local and global explorative random motion towards optimal solutions, and adjusting convergence rate^21^. In this case, the initialization of both *P*_*a*_ and *α* parameters are fixed, hence cannot be amended in the next iterations. Consequently, the main predicament appears in terms of number of iterations required to find optimal solutions. In a case where the value of *P*_*a*_ is small and the value of *α* is large, the performance of the original CS algorithm will be ineffective, which leads to significant increase in number of iterations. In contrast, if the value of *P*_*a*_ is large and the value of *α* is small, the convergence rate will be high (small number of iterations needed) but still unable to find optimal solutions^22^. In this research, an adaptive *P*_*a*_ is introduced to control the convergence rate so that optimal solutions can be found at a reasonable number of iterations. Hence, the dynamic *P*_*a*_ can increase the diversity of solutions and approximation capability.

Second, based on the assumption that a cuckoo lays one egg at a time at a nest of other host bird, the possibility of the egg to survive is quite low. In other words, it is barely for one egg to hatch successfully after being discovered by the host bird. In this regard, there is a need to introduce a dynamic parameter along with the fixed step size, ᵅ for cuckoos to search around the potential good nests (solutions) for laying egg. Precisely, this can been achieved by introducing and embedding an adaptive inertia weight, *w* parameter along with the fixed ᵅ to search for new nests (optimal solutions), *x(t* + 1) via the Lévy flight motion. Hence, cuckoos are capable to explore more rigorously for a better environment if the current habitat is not suitable for breeding.

Third, the assumption is made in the original CS algorithm where the number of available random host nests is fixed. The host birds spot the cuckoos’ eggs with a probability of *P*_*a*_ ∈ [0, 1]. For such incidents, the host birds will either evict the parasitic eggs or abandon the nests totally and seeks for a new site to rebuild the nests^23^. In this case, there is a missing mechanism to select the fittest nests (solutions) with the best probability of cuckoos’ eggs survival. This becomes crucial if the number of host nests is scarce. In this research, a Roulette wheel selection operator is introduced to do an initial stochastic selection of highly potential host nests (solutions).

In this study, all the postulated Pareto-based modified cuckoo search (MCS) algorithms deploy some unique parameters, which are Roulette wheel selection operator, dynamic discovery rate, *P*_*a*_, and the inertia weight, *w* to perform iterative optimizations for rectangular antenna array synthesis. The new solution *x*^(*t*+1)^ for a cuckoo *i*, where the Lévy flight integrated with adaptive weight, *w* is derived as below^24^


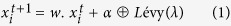


where *α* (must be greater than zero) is the step size related to the scales of the problem of interest and the product ⊕ is the entry-wise multiplication operator. The larger *w* means the greater capability for the proposed algorithms to explore or exploit host nest positions (solutions) and vice versa. Based on (2), *w* is linearly decreased from a relatively large value to a small value through the course so that all the proposed Pareto MCS-based algorithms have a dynamic performance. The mathematical notation is as follow


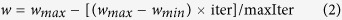


where *w*_*max*_ denotes the maximum weight and *w*_*min*_ denotes the minimum weight, respectively. Besides, the dynamic discovery rate or fraction probability, *P*_*a*_ is also applied, which is calculated as follow


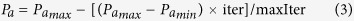


where 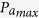 is the maximum discovery rate, and 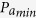 is the minimum discovery rate, respectively.

Recently, CS algorithm has also been prolonged and formulated as multiobjective cuckoo search (MOCS) algorithm to solve multiobjective (MO) optimization problems through Pareto front approach^25^. In this study, the proposed MOCS algorithm non-dominated solutions are validated against a set of MO benchmark test functions, which are Zitzler–Deb–Thiele’s 1 (ZDT 1) and Zitzler–Deb–Thiele’s 3 (ZDT 3) test functions, respectively. The proposed MOCS are then applied to solve structural design nonpolynomial (NP)-hard problems, which are beam design and disc brake design. The simulations for these benchmarks and test functions proved that MOCS algorithm can optimize highly nonlinear problems with complex constraints and diverse Pareto optimal sets^25^.

Principally, this study introduces the implementation of the modified cuckoo search (MCS) algorithm via the global Pareto front approach to determine the three non-dominated alternate solutions, which are optimal positions, excitation amplitudes, and excitation phases of rectangular antenna array elements, respectively. The Pareto front approach has shown to be promising in solving MO optimization problems by accelerating convergence and maintaining a high degree of diversity selected from a set of non-dominated solutions. This study primarily extends the research related to the MO optimization via weighted-sum approach for symmetric linear antenna synthesis^26^ by deploying the strength Pareto trade-offs of three objective functions, *f*_1_, *f*_2_, and *f*_3_. Precisely, the robustness of hybridization of MCS and PSO known as MCSPSO algorithm is found to have a superior performance than the original CS algorithm after undergoing the weighted-sum MO optimization approach^26^ in terms of side lobes suppression, null mitigation, and half-power beamwidth (HPBW) reduction. Alternatively, in this research we put a more concern on the simultaneous trade-off optimization to prevent the possibility of bias among objective functions in weighted sum-approach due to different units or parameters used for each function. Therefore, this research scrutinizes the Pareto front approach to obtain the trade-offs between the three objective functions. The trade-offs feature could give a better observation and comparison of different objective functions performance since many real world applications including electromagnetic (EM) optimization involve conflicting objective functions. In this case, the increase of certain objective function will bring to the decrease of another objective function simultaneously. Consequently, this research focuses specifically on a trade-off optimization of MO functions in which we only observe the objective functions trade-off changes as a whole instead of the individual fitness changes as in weighted-sum approach. Precisely, the objective function, *f*_1_ is defined as


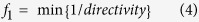


where the directivity of antenna beam solid angle is given by


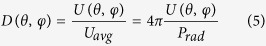


In (5), *U(φ, θ*) = *B*_*o*_*F(φ, θ*) is the antenna radiation intensity per unit solid angle in direction while *U*_*avg*_ is the average antenna radiation intensity or average total power over all directions. The directivity in decibel (dB) unit for an antenna can be measured through a formula


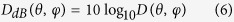


In addition, the fitness function, *f*_2_ is expressed as


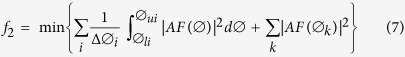


Looking on the right-hand side of (7), the first-term highlights on average SLL suppression whereas the second-term controls prescribed nulls or interferers, respectively. Furthermore, the formula of objective function, *f*_3_ is defined as


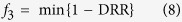


where the dynamic range ratio (DRR) is directly calculated through a formula below


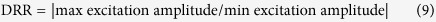


Ideally, the DRR is one where the maximum current amplitude equals to the minimum current amplitude for all 2(*M* × *N*) = 20 × 20 rectangular antenna array isotropic radiators. In order to obtain the Pareto trade-off for *f*_1_, *f*_2_, and *f*_3_, the strength Pareto evolutionary algorithm (SPEA) method is implemented as a basis of the global Pareto front approach to perform the MO optimization for the planar array geometry synthesis. Generally, the concept of Pareto-optimality for a maximization problem with two decision vectors *a, b* ∈ *X* is derived as below^27^


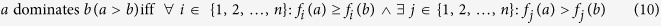



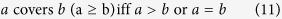


All decision vectors, which are not dominated by any other decision vector in (10) or (11) are called non-dominated or Pareto-optimal. The family of all non-dominated alternate solutions is denoted as Pareto-optimal set (Pareto set) or Pareto-optimal front (Pareto front). It describes the trade-off surface with respect to the *n* objectives^27^. Analogically, for a minimization problem, *a* dominates *b* is written as *a* < *b* whereas *a* covers *b* is denoted as a < b.

In this research, the postulated MCS algorithm is hybridized with the SPEA method forming MCSSPEA to find the Pareto front non-dominated solutions. Furthermore, an integration of this approach together with the hill climbing (HC) stochastic algorithm producing MCSHCSPEA is investigated to improve the local search capability. Finally, we further assess the performance of evolutionary search by incorporating the PSO metaheuristic algorithm into MCS and SPEA, also known as MCSPSOSPEA to analyze the robustness of hybridization. The MCSSPEA, MCSHCSPEA, and MCSPSOSPEA hybrid algorithms deploy exhaustively the dynamic discovery rate, *P*_*a*_, and the inertia weight, *w*, simultaneously. In this case, the dynamic *P*_*a*_ reduces the possibility of host birds of other species to discover the cuckoo’s egg as the iteration increases. Precisely, the dynamic discovery rate is getting smaller gradually as the number of iteration rises initiating the brood-parasitism behavior successes as defined in (3). Moreover, the dynamic inertia weight, *w* is calculated using (2). There is a mechanism for MCSSPEA, MCSHCSPEA, and MCSPSOSPEA optimizers to increase the spread of Pareto front to reduce the local trap predicament as sometimes appeared in the original SPEA. The distance for three MO functions with *p* non-dominated solutions is calculated as below


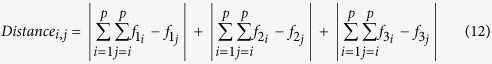


The following eqs (13),(14),(15), show the Pareto front spread fitness formulation of *p* non-dominated solutions^28^


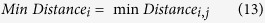



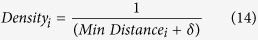



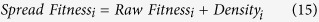


Equation (14) shows that the smaller the distances between the inter-Pareto raw points, the higher the density would be. The denominator of (14) has the minimum distance added with a constant value, *δ* to ensure the density is enough to spread the raw points as calculated in (15). The setting and formulation is desired to achieve the big spread of optimal non-dominated solutions in the Pareto front trade-offs search domain. The *RawFitness*_*i*_ in (15) is derived originally from the *f*_1_, *f*_2_, and *f*_3_ values of the strength Pareto front. Since this study involves Pareto-based multiobjective (MO) optimization, hypervolume indicator or hypervolume measure of the dominated portion of the objective space is applied as a quality measure for Pareto set approximations. In this analysis, the hypervolume indicator *I*_*H*_(*A*) of a solution set *A* ⊆ *X* can be defined as the hypervolume of the space with 3 objective functions that are dominated by the set *A* and is bounded by a reference point *r* = (*r*_1_, *r*_2_,.*r*_3_) 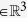 defined by^29^


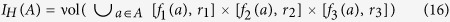


where νol(.) is stated as the Lebesgue measure and 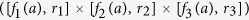 is the *k*-dimensional hypercuboid (where *k* = 3 in this study) consisting of all Pareto front points, which are weakly dominated by the point *a* but not weakly dominated by the reference point.

**Figure 1:**
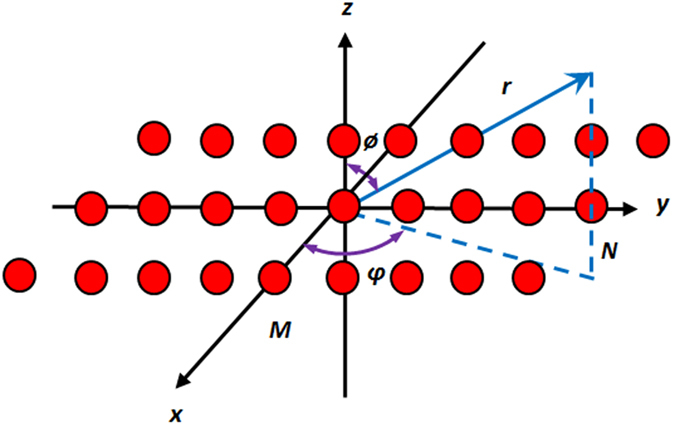
Geometry of 2(*M* × *N*) Rectangular Antenna Arrays. In this experiment, we deploy the inter-element spacing with respect to the *λ*/2 distance of both the *m* and *n*-th elements to avoid mutual coupling occurs in the rectangular antenna array.

In this experiment, we assign the 2(*M* × *N*) array elements or isotropic radiators on *xy*-plane symmetrically as depicted in Fig. 1. The *M* refers to the number of radiators located at the *x*-axis whereas the *N* signifies the number of radiators located at the *y*-axis, respectively. In this case, for uniformity, both *M* and *N* are assumed to be equal in terms of number of quantity and inter-element distance. For 2(*M* × *N*) = 20 × 20 planar antenna, it means that each *x* and *y*-axis has 20 symmetric array elements. The array factor (*AF*) for the azimuth plane is formulated as


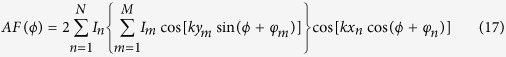


where *k* (equals to 2*π/λ*) is the calculated wave number, and *I*_*m*_, *I*_*n*_, *φ*_*m*_, *φ*_*n*_, *x*_*m*_, and *x*_*n*_ are the excitation amplitude, excitation phase, and location of both the *m* and *n*−th array elements on *xy*-plane, respectively. Based on (17), the MCS MO algorithm is postulated to optimize *I*_*m*_, *I*_*n*_, *φ*_*m*_, *φ*_*n*_, *x*_*m*_, and *x*_*n*_ values of the rectangular antenna array elements with minimum average SLL, HPBW and significant nulls mitigation.

### Proposed MCS Algorithms via Global Pareto Front Approach

Below is the postulated pseudo-code of MCSPSOSPEA algorithm, which is validated and simulated in this study:

#### begin

Let *iter* denote the iteration no. of MCSPSOSPEA.

iter ← 1;

Init. pop. of host nests with size *n* at *iter*=1;

***for*** each iteration

Operate the Roulette wheel selection to obtain the “fittest” host nests with size *n*;

Generate a new set of solutions (host nests) but keep the Current best (say, *i*) randomly by Lévy flights incorporating with inertia weight, *w*, which controls the search ability according to (1);

Evaluate new solution MO fitness, *f*_*i*_ according to (4), (7), and (8);

Get a selected set of host nests among n (say, *j*) and calculate its MO fitness, *f*_*j*_ according to (4), (7), and (8);

***if*** (f_i ≤ _f_j_) % fitness minimization %

Replace *j* by the new set of solutions, *i*;

**end**

A dynamic fraction probability, *P*_*a*_ of worse nests is abandoned and a new nest (set of solution) is built;

Keep the best nests with quality solutions;

Let the best nests become as initial particles;

***for*** each particle

Calculate MO fitness value according to (4), (7), and (8);

***if*** the fitness value is better than the best MO fitness value (*pbest*) in history

Set current value as the new *pbest*;

***end***

***end***

***for*** each particle

Calculate particle velocity;

Update particle position;

***end***

Evaluate the updated current MO fitness value according to (4), (7), and (8);

***if*** the new current MO fitness value is better than the fitness of *pbest*;

Set current value as the new *pbest*;

***end***

Keep the best particles with quality solutions;

Rank the solutions and find the current best particle;

Population ← Current best particle;

Archive ← Ø;

***for** S*_*i*_∈ Population


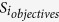
 ← CalculateObjectives (*S*_*i*_);

***end***

Union ← Population + Archive;

***for*** 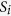 ∈ Union


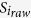
 ← CalculateRawFitness (*S*_*i*_, Union);


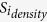
 ← CalculateSolutionDensity (*S*_*i*_, Union);


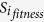
 ← 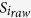 + 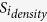;

***end***

Archive ← GetNonDominated (Union);

***if*** Size (Archive) < *Archive*_*size*_

PopulateWithRemainingBest (Union, Archive, *Archive*_*size*_);

***elseif*** Size (Archive) > Archive_size_

RemoveMostSimilar (Archive, *Archive*_*size*_);

***end***

Return (GetNonDominated (Archive));

***end***

Post–process result (Archive) and visualization;

***end***

On the other hand, the following is the proposed pseudo-code for the MCSHCSPEA hybrid algorithm, which is also developed and validated in this study:

***begin***

Let *iter* denote the iteration no. of MCSHCSPEA.

*iter* ← 1;

Init. pop. of host nests with size *n* at *iter* = 1;

***for*** each iteration

Operate the Roulette wheel selection to obtain the “fittest” host nests with size *n*;

Generate a new set of solutions (host nests) but keep the Current best (say, *i*) randomly by Lévy flights incorporating with inertia weight, *w*, which controls the search ability according to (1);

Evaluate new solution MO fitness, *f*_*i*_ according to (4), (7), and (8);

Get a selected set of host nests among n (say, *j*) and calculate its MO fitness, *f*_*j*_ according to (4), (7), and (8);

***if** (f*_*i*_ ≤ *f*_*j*_) % *fitness minimization* %

Replace *j* by the new set of solutions, *i*;

***end***

A dynamic fraction probability, *P*_*a*_ of worse nests is abandoned and a new nest (set of solution) is built;

Keep the best nests with quality solutions;

Let the best nests become as initial particles;

*X*’ ← perturbation (*x*)

***for*** each individual, *x*’

Calculate MO fitness, *fx*’ value according to (4), (7), and (8);

***end***

***if** (fx*’ ≤ *fx*) % fitness minimization%

Replace x by the new set of solutions, *x*’;

***end***

Keep the best individuals with quality solutions;

Rank the solutions;

Population ← Current best individual;

Archive ← Ø;

***for** S*_*i*_ ∈ Population


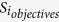
 ← CalculateObjectives (*S*_*i*_);

***end***

Union ← Population + Archive;

***for** S*_*i*_ ∈ Union


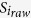
 ← CalculateRawFitness (*S*_*i*_, Union);


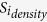
 ← CalculateSolutionDensity (*S*_*i*_, Union);


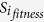
 ← 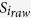 + 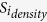;

***end***

Archive ← GetNonDominated(Union);

***if*** Size(Archive) < *Archive*_*size*_

PopulateWithRemainingBest (Union, Archive, *Archive*_*size*_);

*elseif* Size (Archive) > *Archive*_*size*_

RemoveMostSimilar (Archive, *Archive*_*size*_));

***end***

Return (GetNonDominated (Archive));

***end***

Post–process result (Archive) and visualization;

***end***.

## Results

### Simulation of the Proposed Algorithms on Both the ZDT1 and ZDT3 Test Functions

In the first stage, we evaluated the proposed MCSSPEA, MCSHCSPEA and MCSPSOSPEA along with the original SPEA to perform MO Pareto optimization using the ZDT1 and ZDT3 benchmark test functions. In this experiment, we simulated ten evaluations with 1000 iterations of optimization for each evaluation to ensure the consistency of the Pareto trade-offs for minimization of MO in this case *f*_1_ and *f*_2_ defined as^30^


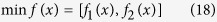


when *f*_1_ and *f*_2_ reach the optimal simultaneously, the optimal solution will become Pareto non-dominated solutions^30^.

The convex Pareto-optimal front ZDT1 objective functions with the domain [0, 1] are defined as^30^,^31^


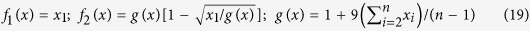


The ZDT3 function adds a discreteness feature to the Pareto-optimal front that consists of few noncontiguous convex parts. The introduction of sine function causes discontinuities in the front not in the parameter space. The objective functions in Pareto front ZDT3 with the domain [0, 1] are defined as^30^,^31^


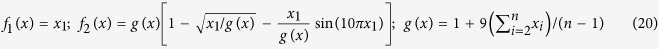


**Figure 2:**
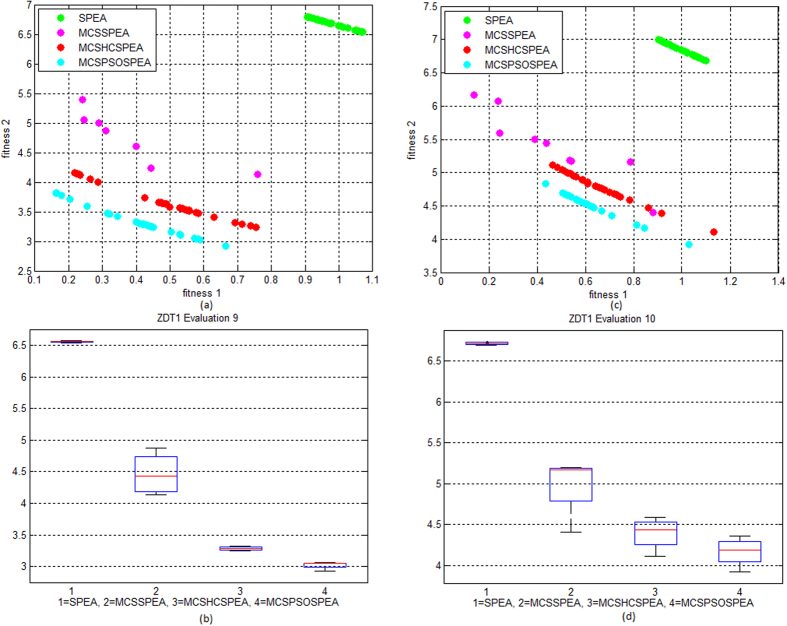
Strength Pareto Evolutionary Algorithm Front Approximations on ZDT1 Test Function (10 evaluations, maxIter = 1000). (**a**) The verification and comparison of the proposed MCSSPEA, MCSHCSPEA, and MCSPSOSPEA with the standard SPEA in evaluation no. 9. (**b**) The verification and comparison of the proposed MCSSPEA, MCSHCSPEA, and MCSPSOSPEA with the standard SPEA in evaluation no. 10. (**c**) We map the trade-off data distribution in boxplot for evaluation no. 9. (**d**) We map the trade-off data distribution in boxplot for evaluation no. 10.

**Figure 3:**
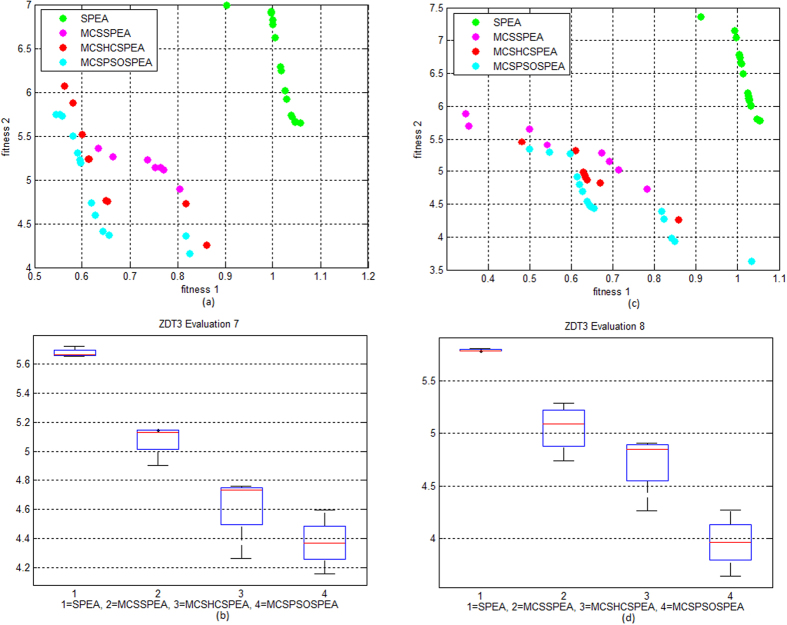
Strength Pareto Evolutionary Algorithm Front Approximations on ZDT3 Test Function (10 evaluations, maxIter = 1000). (**a**) The verification and comparison of the proposed MCSSPEA, MCSHCSPEA, and MCSPSOSPEA with the standard SPEA in evaluation no. 7. (**b**) The verification and comparison of the proposed MCSSPEA, MCSHCSPEA, and MCSPSOSPEA with the standard SPEA in evaluation no. 8. (**c**) We map the trade-off data distribution in boxplot for evaluation no. 7. (**d**) We map the trade-off data distribution in boxplot for evaluation no. 8.

Figure 2a to d shows the Pareto trade-off and boxplot of the proposed algorithms testing on convex ZDT1 functions for evaluation number 9 and 10, respectively. The proposed MCSPSOSPEA clearly outperformed other competitors including the original SPEA by having the most minimum Pareto-optimal front. Statistically, the boxplot shows that the MCSPSOSPEA has the smallest minimum, 1^st^ quartile, median, 3^rd^ quartile, and maximum convergence values of distributed solutions. On the other hand, Fig. 3a to d shows the Pareto trade-off and boxplot of the proposed algorithms testing on the ZDT3 functions for evaluation number 7 and 8, respectively. Once again, the MCSPSOSPEA surpassed other rivals with the most minimum Pareto-optimal front. The boxplot displays that the MCSPSOSPEA had the smallest minimum, 1^st^ quartile, median, 3^rd^ quartile, and maximum convergence values of distributed solutions. Overall, the proposed MCSPSOSPEA, MCSHCSPEA, and MCSSPEA visibly had smaller convergence values than the standard SPEA in MO Pareto optimal front of ZDT1 and ZDT3 test functions, respectively.

### Simulation for 2(*M* × *N*) = 20 × 20 Rectangular Array under the Dolph-Chebyshev Window

In the second stage of the global Pareto MO simulation, the postulated MCSPSOSPEA, MCSHCSPEA, and MCSSPEA using Mantegna’s algorithm as the *α*–stable distribution method constrained by host nest (population) = 20, length step factor = *L*/100 or 0.01, and *α *=* *2.0 (Lévy flight Gaussian distribution) were synthesized and verified on the 2(*M* × *N*) = 20 × 20 rectangular antenna array. In this case, the proposed MCSSPEA, MCSHCSPEA and MCSPSOSPEA optimizers with the dynamic inertia weight, *w* magnitude domain of [0.80 1.20] were directly compared with the standard SPEA and conventional arrays in the Dolph-Chebyshev signal processing window with the relative SLL, *R* = −30 dB for array elements on each of *x* and *y*-axis. The bigger, *w* magnitude domain leads the MCS algorithms to gain a more control on the Lévy flight motions with a heavy-tailed and *α*-stable distribution towards the best host nest (candidate solution) in search space. The MCSPSOSPEA optimizer applied the particle swarm optimization (PSO) algorithm restricted by the dynamic random particle velocity domain of [−0.1 + 0.1]. All the proposed algorithm source codes were composed using the MATLAB 7.14 (R2012a) software and executed via a notebook with the Intel^®^ Core ™ i5-3210 M (X64−based processor) operating at 2.50 GHz processing cycle and deploying 4.00 GB of random access memory (RAM). In this test precisely, the MATLAB simulation executed 1000 iterations of strength Pareto optimization to find the set of three non-dominated solutions simultaneously, which were rectangular or planar array elements locations, excitation amplitudes, and excitation phases, respectively.

**Figure 4:**
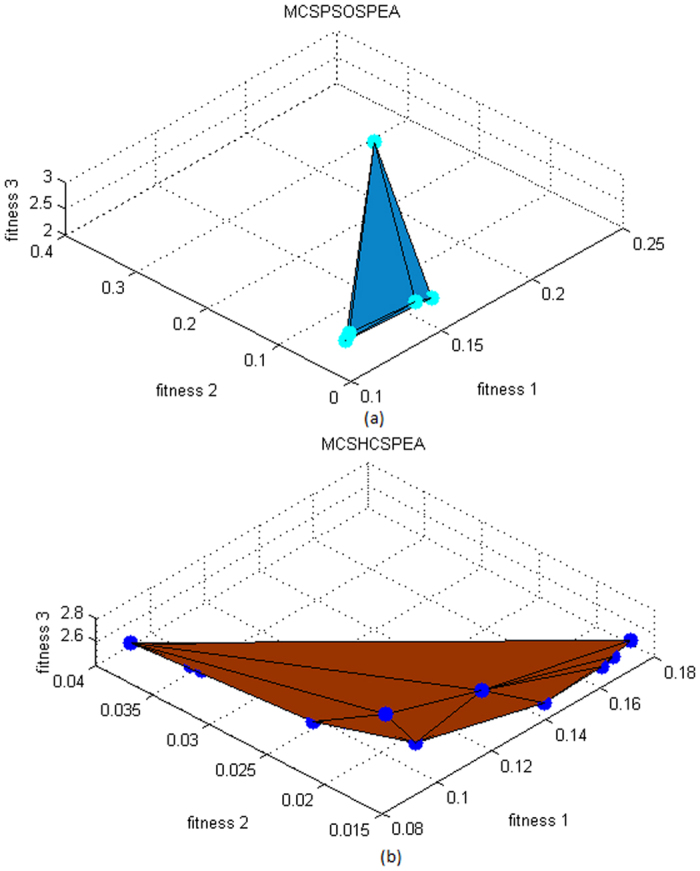
Strength Pareto Evolutionary Algorithm Front Approximations 2(*M* × *N*) = 20 × 20, Dolph-Chebyshev, maxIter = 1000). (**a**) The three objective functions trade**-**off simulation for the MCSPSOSPEA optimizer. (**b**) The three objective functions trade-off simulation for the MCSHCSPEA optimizer.

Overall, the spread Pareto fitness domain for all optimizers were *f*_1_ ∈ [0.08, 0.24], *f*_2_ ∈ [0.012, 0.040], and *f*_3_ ∈ [2.0, 3.0], respectively. Figure 4a and b show the three-dimensional (3D) Pareto fronts plot for MCSPSOSPEA and MCSHCSPEA, respectively. Precisely, we calculated the hypervolume of the Pareto front 3D plot for all the tested optimizers. Ideally, the hypervolume closed to zero was preferred for a global Pareto minimization process. In the simulation, the MCSPSOSPEA had the hypervolume of 4.0000 × 10^−3^ unit^3^, the MCSHCSPEA had the hypervolume of 2.1894 × 10^−4^ unit^3^, and each of the MCSSPEA and SPEA optimizers had the hypervolume of 2.6352 × 10^−4^ unit^3^, respectively. Looking at the Pareto trade-offs aspect, the MCSPSOSPEA has some Pareto optimal points that were dominated by MCSHCSPEA, which had the smallest hypervolume. In sum, every tested SPEA-based optimizer had a very small hypervolume, which closed to zero after reaching the maximum iteration. Moreover, all the strength Pareto optimizers were almost similar with the estimated differences less than 3.8000 × 10^−3^ unit^3^.

**Figure 5:**
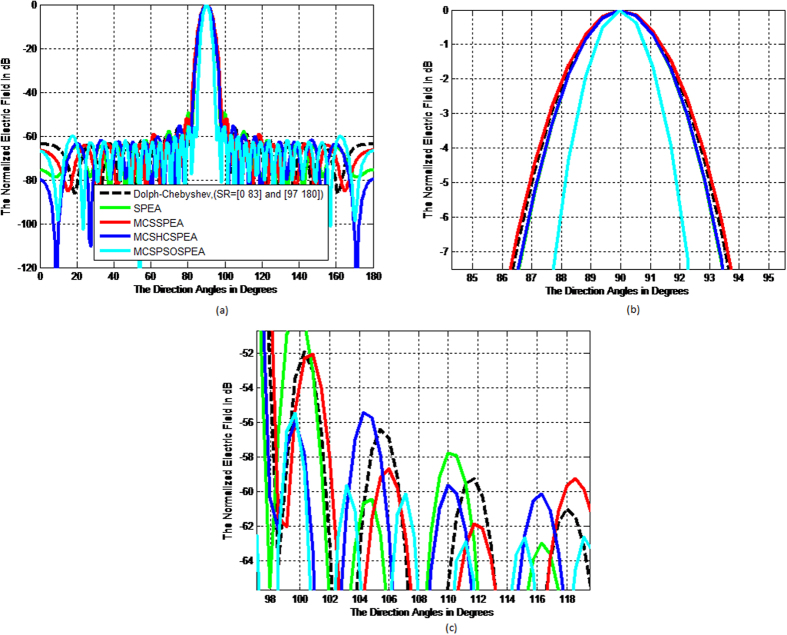
Normalized Pattern for SPEA-based Rectangular Arrays 2(*M* × *N*) = 20 × 20, Dolph-Chebyshev, maxIter = 1000). (**a**) Overall normalized radiation pattern. (**b**) Half–power beamwidth pattern. (**c**) Average side lobe level pattern.

**Figure 6:**
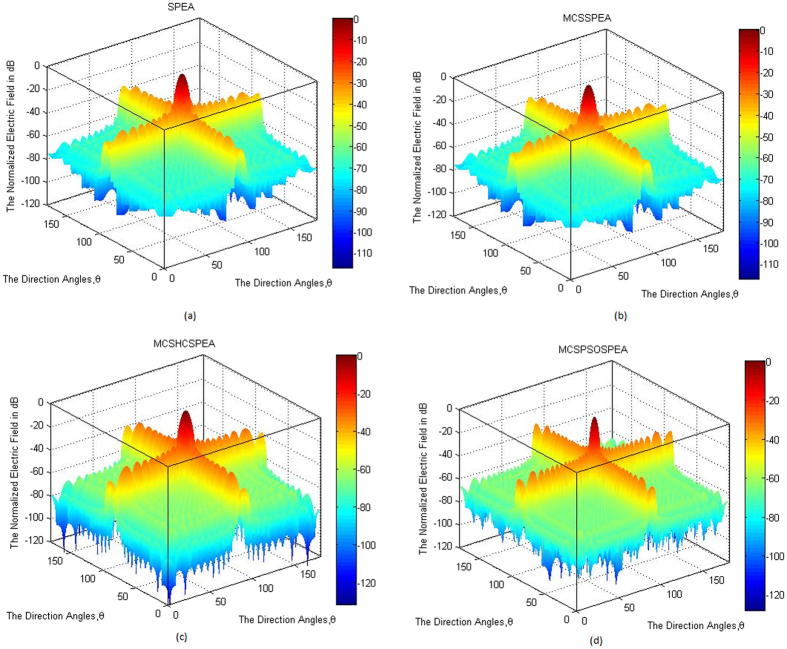
3D Normalized Antenna Pattern for SPEA-based Rectangular Arrays 2(*M* × *N*) = 20 × 20, Dolph-Chebyshev, maxIter = 1000). We simulate four SPEA-based rectangular arrays, which are (**a**) Original SPEA. (**b**) Hybrid MCSSPEA. (**c**) Hybrid MCSHCSPEA. (**d**) Hybrid MCSPSOSPEA.

Based on Fig. 5a, the overall normalized radiation pattern for the hypothesized MCSPSOSPEA optimizer evidently outperformed other optimizers through executing the substantial low average SLL suppression and highest directivity of the main beam, simultaneously. Mathematically, the half-power beamwidth (HPBW) is defined as the angular separation where the magnitude of the normalized radiation pattern decreased by 50% (or −3 dB) relatively from the peak of the main beam. Based on Fig. 5b, the MCSPSOSPEA optimizer formed the radiation pattern significantly decreased to −3 dB at 88.5537° and 91.4476° with the HPBW of 91.4476°−88.5537° = 2.8939°. Figure 3b also depicts the MCSPSOSPEA-based array generated the highest directivity measurement of 9.3810 dB. This is followed by the MCSHCSPEA counterpart with the directivity of 7.7416 dB. Moreover, the postulated MCSPSOSPEA optimizer had the slightly best average SLL suppression between 0.7173 dB and 4.1547 dB lower than the conventional array within the suppression domains of [45° 85°] and [95° 135°] as shown in Fig. 5a and c, respectively. Moreover, Fig. 6a till 6d display the three-dimensional (3D) normalized radiation plot for all the tested SPEA-based rectangular array optimizers. Graphically, the MCSPSOSPEA optimizer generated relatively the narrowest main lobe due to the smallest HPBW and highest directivity. Moreover, the MCSPSOSPEA technique also executed the thinnest main and side lobes compared to other tested SPEA optimizers.

**Figure 7:**
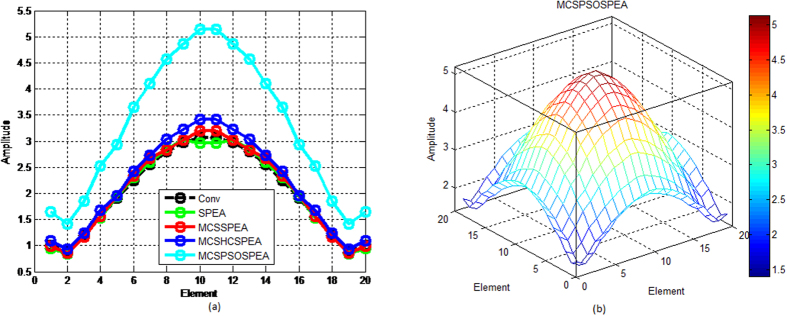
Optimal Excitation Amplitude for SPEA-based Rectangular Arrays 2(*M* × *N*) = 20 × 20, Dolph-Chebyshev, maxIter = 1000). (**a**) We compare four SPEA-based and conventional rectangular antenna arrays excitation amplitude in 2D plot. (**b**) The proposed MCSPSOSPEA rectangular array excitation amplitude in 3D plot.

**Figure 8:**
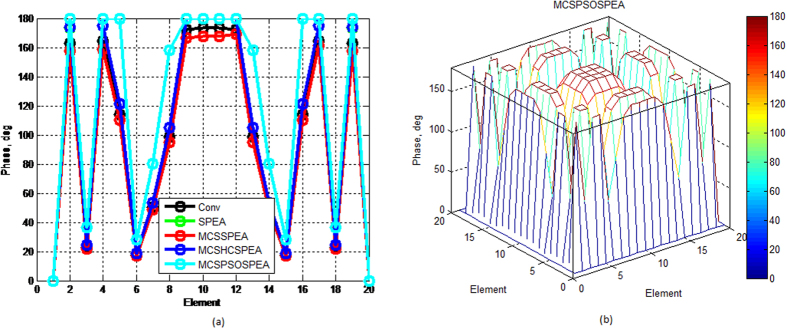
Optimal Excitation Phase for SPEA-based Rectangular Arrays 2(*M* × *N*) = 20 × 20, Dolph–Chebyshev, maxIter = 1000). (**a**) We compare four SPEA-based and conventional rectangular antenna arrays excitation phase in 2D plot. (**b**) The proposed MCSPSOSPEA rectangular array excitation phase in 3D plot.

Figure 7a and b show that compared to other opponents, the proposed MCSPSOSPEA optimizer deviated furthest its Dolph-Chebyshev current amplitude with respect to the conventional array. Furthermore, it also had the biggest optimal excitation phase deviations compared to other rivals as depicted in Fig. 8a and b. All of these findings become the key factor for the MCSPSOSPEA optimizer using the selected Pareto optimal solutions to design a rectangular antenna array, which can suppress the lowest average side lobes while enhancing the main beam intensity. In fact, the MCSPSOSPEA has better global Pareto front trade-offs specifically with the smallest *f*_1_ and *f*_3_ values despite having a slightly bigger Pareto front 3D plot hypervolume. As a result, the MCSPSOSPEA produced the highest antenna directivity, smallest HPBW, and lowest average SLL, respectively.

In this experiment, the proposed MCSPSOSPEA generated 6 non-dominated solutions whereas the MCSHCSPEA counterpart produced 14 non-dominated solutions, respectively. Table 1 shows the selected fitness trade-off values for comparing the tested Pareto algorithms demonstrated in Fig. 5 till 8. Precisely, the MCSPSOSPEA had the smallest relative global Pareto front values with respect to *f*_1_ and *f*_3_ after 1000 iterations. As enlisted in Table 2, the proposed MCSPSOSPEA algorithm executed the largest optimal location deviations (with respect to *λ*/2) compared to the conventional array in both *x* and *y*-axes. Precisely, the MCSPSOSPEA had the deviations between |±0.3026| and |±5.7494| for all 2 *N* = 20 rectangular array elements. Practically, MCSPSOSPEA planar antenna had the drawback where its largest optimal inter-element distance led to the biggest aperture size. Furthermore, Tables 1 and 2 also show that both SPEA and MCSHCSPEA were almost similar (the selected fitness differences were less than 0.01 and location differences were less than ±0.01 between each other). This was mainly because the non-dominated points (optimal solutions) of both algorithms were chosen closer to each other in the Pareto front. Table 3 shows that the MCSPSOSPEA hybrid algorithm also attained the biggest optimal amplitude deviations in regard to the conventional array. In this case, the MCSPSOSPEA had the deviations between 0.5296 and 2.0655. Based on Table 3, the MCSPSOSPEA planar antenna also had another downside where it consumed relatively the most power (highest feed current amplitude) applied on isotropic radiators. Besides, Table 4 shows the postulated MCSPSOSPEA also produced the largest optimal phase variations in regard to the conventional array with the variations between 0° and 66.1753°, respectively. In this case, the proposed MCSPSOSPEA-based optimizer had the widest phase domain of [0° 180°] for all 2 *N* = 20 rectangular array elements.

### Simulation for 2(*M* × *N*) = 20 × 20 Rectangular Array with Predefined Nulls under the Uniform Window

In the third stage, a more extensive simulation was done on the 2 *M* × *N* = 20 × 20 rectangular broadside antenna array with a main beam steered at 90° along with four prescribed interferers occurred at 55°, 60°, 120°, and 125°, respectively. In this simulation, the MCSPSOSPEA, MCSHCSPEA, and MCSSPEA optimizers applied the Mantegna’s *α*-stable distribution method with setting internal parameters of host nest or population = 20, length step factor = *L*/100 or 0.01, and *α *=* *2.0 (Lévy flight Gaussian distribution). Besides, all the proposed MCS-based optimizers were bounded by a dynamic *P*_*a*_ amount within the domain of [0.01 0.25] and a dynamic *w* amount within the domain of [0.80 1.20] to control the exploration and exploitation of optimal solutions within search space. The postulated MCSPSOSPEA optimizer deployed the standard PSO algorithm limited by the dynamic random particle velocity domain of [−0.1 + 0.1].

**Figure 9:**
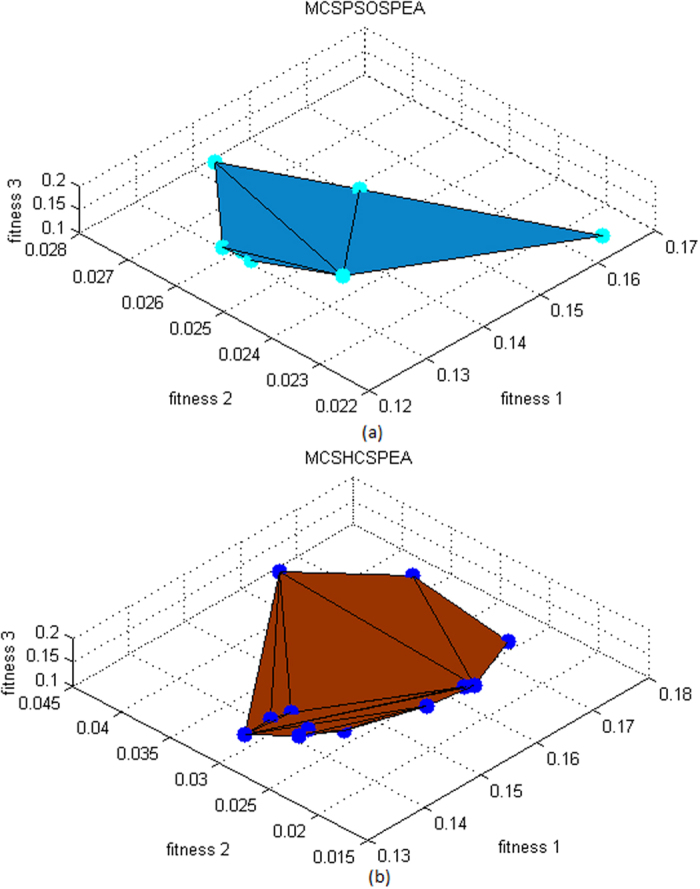
Strength Pareto Evolutionary Algorithm Front Approximations 2(*M* × *N*) = 20 × 20, Uniform, Null = [55°, 60°, 120°, 125°], maxIter = 1000). (**a**) The three objective functions trade-off simulation for the proposed MCSPSOSPEA optimizer. (**b**) The three objective functions trade-off simulation for the proposed MCSHCSPEA optimizer.

After executing 1000 iterations, the MO trade-off domains for all Pareto optimizers became *f*_1_ ∈ [0.12, 0.18], *f*_2_ ∈ [0.015, 0.045], and *f*_3_ ∈ [0.0, 0.2], respectively. Fig. 9a and b depicts the 3D Pareto fronts plot for MCSPSOSPEA and MCSHCSPEA, respectively. Precisely, the original SPEA had the smallest hypervolume of 5.9083 × 10^−6^ unit^3^ whereas the postulated MCSPSOSPEA had the hypervolume of 7.9002 × 10^−6^ unit^3^ followed by the MCSSPEA counterpart with the hypervolume of 2.1662 × 10^−5^ unit^3^, and the MCSHCSPEA algorithm with the hypervolume of 2.3462 × 10^−5^ unit^3^, respectively. Generally, all the hypervolume values were so small (near to zero) due to the strength Pareto fitness minimization process, and were almost similar with the estimated differences less than 1.8000 × 10^−5^ unit^3^.

**Figure 10:**
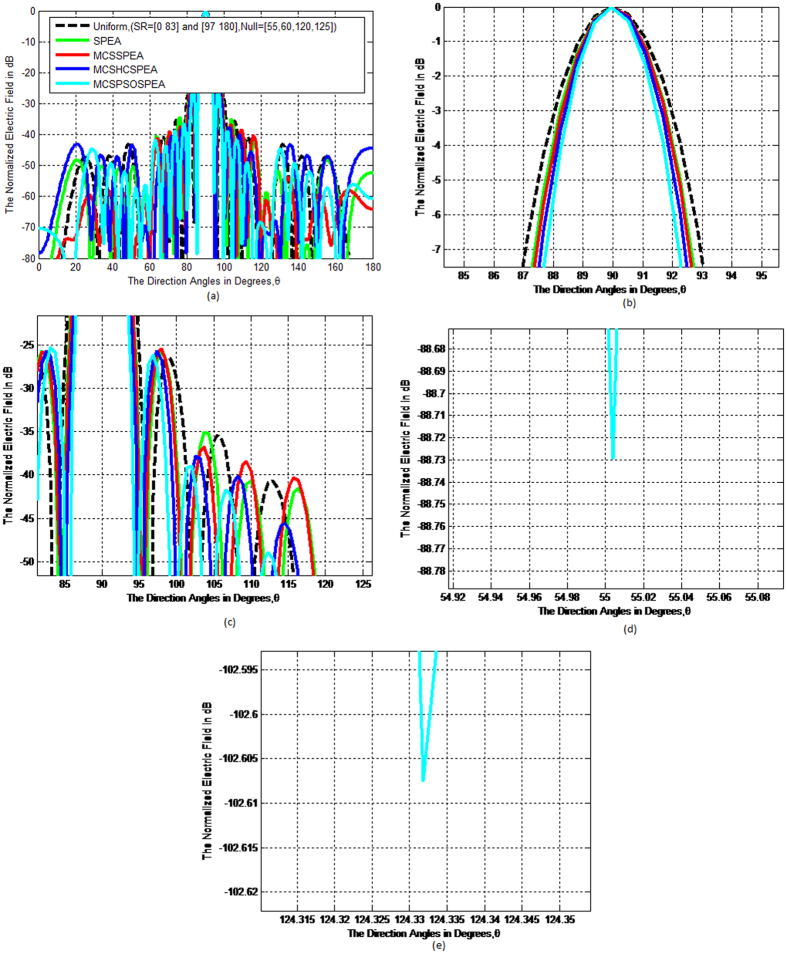
Normalized Pattern for SPEA-based Rectangular Arrays 2(*M* × *N*) = 20 × 20, Uniform, Predefined Null = [55°, 60°, 120°, 125°], maxIter = 1000). (**a**) Overall normalized radiation pattern. (**b**) Half-power beamwidth pattern. (**c**) Average side lobe level pattern. (**d**) Null mitigation between 55° and 60°. (**e**) Null mitigation between 120° and 125°.

Figure 10a shows that the MCSPSOSPEA-based array produced the best SLL suppression particularly compared to other rectangular antenna arrays between the [50° 85°] and [95° 130°] regions, respectively. Figure 10a and c clearly shows that the MCSPSOSPEA-based array yielded the average SLL between 1.0300 and 9.7884 dB below the conventional array, respectively. Based on Fig. 10b, the MCSPSOSPEA-based array also produced the highest main beam intensity due to the smallest HPBW of 91.4382°−88.5093° = 2.9289° and the highest planar array directivity of 7.7381 dB, respectively. This was followed by the MCSHCSPEA counterpart that had the larger HPBW of 91.6171°−88.3928° = 3.2243°, and the smaller directivity of 7.2534 dB, respectively. Based on Fig. 10d, the proposed MCSPSOSPEA optimizer executed the significant predefined null mitigation of −88.7293 dB nearly at direction of arrival (DoA) of 55.0039°. Similarly, Fig. 10e indicates that the MCSPSOSPEA optimizer also had the remarkable predefined null mitigation of −102.6074 dB around at DoA of 124.3318°. Overall, the proposed MCSPSOSPEA algorithm surpassed the MCSPSO counterpart applied in the weighted-sum MO optimization approach^26^ for 2 *N* = 20 linear antenna array, which had the average SLL between 0.047 and 3.826 dB lower than the conventional array, HPBW of 92°−88° = 4°, and maximum null mitigation of −70.661 dB, respectively. Consequently, this shows that Pareto MO optimization yielded better performance indicator than the weighted-sum approach using the same objective functions.

**Figure 11:**
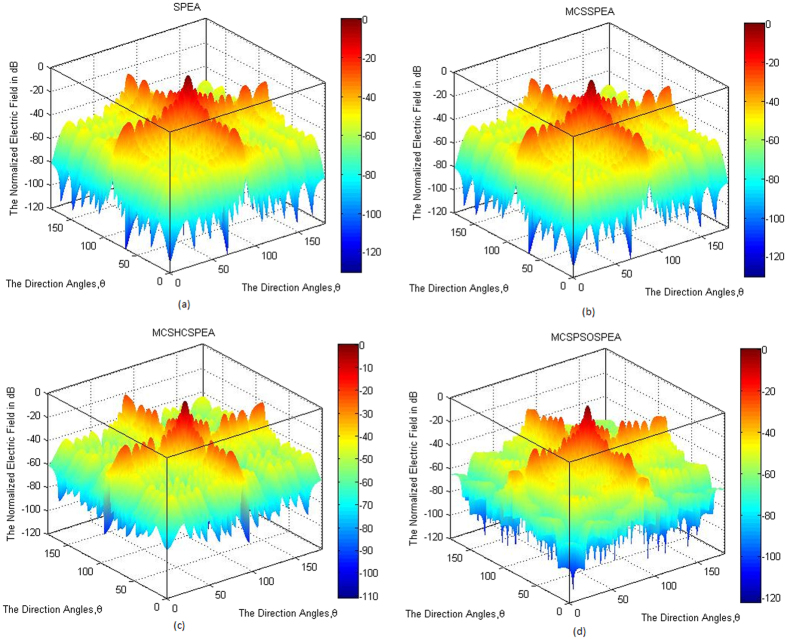
3D Normalized Antenna Pattern for SPEA-based Rectangular Arrays 2(*M* × *N*) = 20 × 20, Uniform, Null = [55°, 60°, 120°, 125°], maxIter = 1000). We simulate 4 SPEA-based rectangular arrays, which are (**a**) Original SPEA. (**b**) Hybrid MCSSPEA. (**c**) Hybrid MCSHCSPEA. (**d**) Hybrid MCSPSOSPEA.

Figure 11a to d show the 3D normalized radiation plot for all the tested standard and enhanced SPEA-based rectangular array optimizers. All the optimizers generated their respective normalized radiation patterns symmetrical on the *xy*-plane. Once again, the MCSPSOSPEA optimizer emitted relatively the narrowest main beam with the smallest HPBW and highest directivity, simultaneously. Moreover, both the MCSPSOSPEA and MCSHCSPEA had the main and side lobes slightly slimmer than the standard SPEA and MCSSPEA counterparts, respectively.

**Figure 12:**
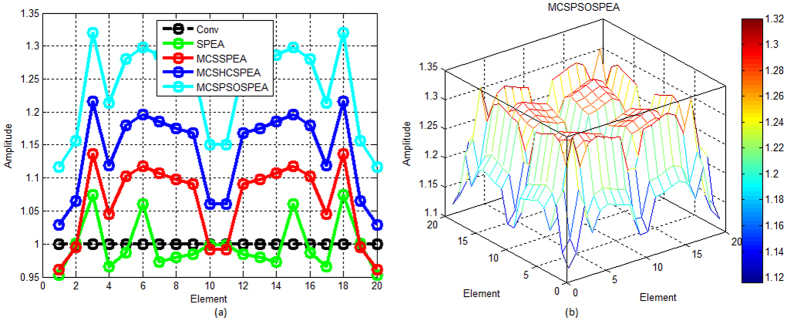
Optimal Excitation Amplitude for SPEA-based Rectangular Arrays 2(*M* × *N*) = 20 × 20, Uniform, Null = [55°, 60°, 120°, 125°], maxIter = 1000). (**a**) We compare four SPEA-based and conventional rectangular antenna arrays excitation amplitude in 2D plot. (**b**) The proposed MCSPSOSPEA rectangular array excitation amplitude in 3D plot.

**Figure 13:**
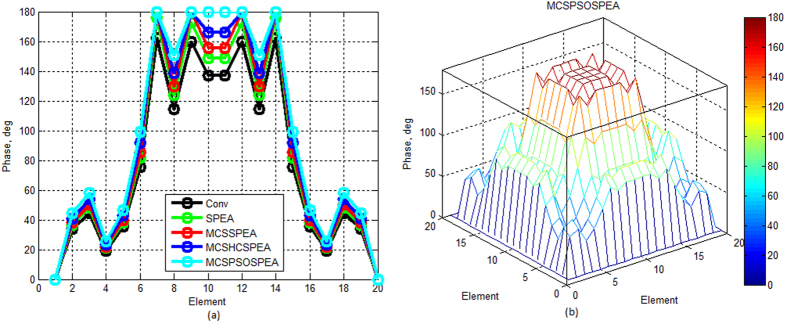
Optimal Excitation Phase for SPEA-based Rectangular Arrays 2(*M* × *N*) = 20 × 20, Uniform, Null = [55°, 60°, 120°, 125°], maxIter = 1000). (**a**) We compare four SPEA-based and conventional rectangular antenna arrays excitation phase in 2D plot. (**b**) The proposed MCSPSOSPEA rectangular array excitation phase in 3D plot.

Based on Fig. 12a and b, the proposed MCSPSOSPEA optimizer yielded the biggest relative optimal excitation amplitude deviations with respect to the conventional array for all 2(*M* × *N*) = 20 × 20 rectangular antenna array elements. This was followed by the MCSHCSPEA, MCSSPEA, and original SPEA counterparts, correspondingly. Moreover, the MCSPSOSPEA optimizer also produced slightly the biggest optimal excitation phase variations compared to the conventional array as shown in Fig. 13a and b. These significant variations were the key factors for the MCSPSOSPEA algorithm using the selected non-dominated solutions to suppress the lowest average SLL while preserving the main lobe and mitigating significantly prescribed nulls. Using the selected non-dominated solutions, the MCSPSOSPEA algorithm had a better strength Pareto trade-off with the smallest *f*_1_ and *f*_3_ values despite having a slightly bigger hypervolume of Pareto front 3D plot than the standard SPEA rival. Thus, the MCSPSOSPEA optimizer generated the highest rectangular antenna directivity, smallest HPBW, lowest average SLL, and best predefined nulls mitigation, simultaneously.

In this simulation, the postulated MCSPSOSPEA generated 10 non-dominated solutions whereas the MCSHCSPEA counterpart produced 13 non-dominated solutions, respectively. Table 5 enlists the fitness of each objective function according to the selected non-dominated solutions for the comparison of all the tested Pareto algorithms as shown in Figs 8 to 11, respectively. Precisely, the MCSPSOSPEA had the smallest relative global Pareto front values with respect to *f*_1_ and *f*_3_. Moreover, Table 6 shows that the MCSPSOSPEA hybrid optimizer ensured the biggest relative optimal location variations between |±0.1578| and |±2.9991| in regard to the conventional array for all 2(*M* × *N*) = 20 × 20 rectangular antenna array isotropic radiators. Similarly, the MCSPSOSPEA optimizer also warranted the biggest relative optimal amplitude variations between 0.1162 and 0.3200 in regard to the conventional array for all 2(*M* × *N*) = 20 × 20 rectangular antenna array elements as presented in Table 7. Based on Tables 6 and 7, the MCSPSOSPEA planar antenna was optimally designed and implemented with the relatively biggest aperture size and highest feed current amplitude applied for all array elements. Moreover, the MCSPSOSPEA hybrid optimizer also produced the biggest relative optimal phase variations between 0° and 42.8517° with respect to the conventional array as clearly shown in Table 8. There was an element with the excitation phase of 0° and three elements with the excitation phase of 180°, respectively. In brief, the hypothesized MCSPSOSPEA could search further (at the maximum or minimum extent) the optimal locations, current amplitudes and current phases hence, improved the rectangular antenna array main beam scanning and predefined nulls mitigation.

## Conclusion

Overall, the proposed MCSPSOSPEA, MCSHCSPEA, and MCSSPEA are proven to produce better global Pareto fronts than the standard SPEA in ZDT1 and ZDT3 test functions as well as in rectangular antenna array synthesis. In this study, the proposed MCSPSOSPEA is clearly the best Pareto MO metaheuristic optimizer. The hybrid MCSPSOSPEA is capable to enhance the exploration of potential best host nests or Pareto optimal solutions within 3-dimensional search space to produce a better diversity of non-dominated solutions signifying optimal element locations, excitation amplitudes, and excitation phases, respectively. The introduction of some value-added optimizer attributes, e.g. Roulette wheel selection operator to choose the initial host nests that give better results, adaptive weight, *w* to control the positions exploration of the potential best host nests, dynamic discovery rate, *P*_*a*_ to manage the fraction probability of finding the best host nests within search space, and particle velocity and position iterative updating procedures in the PSO optimizer have become primarily the key factors. These valued-added attributes support the proposed MCSPSOSPEA to perform the Lévy flight searching motion effectively in locating the potential best host nests, which then become as the best Pareto non-dominated solutions. Precisely, the proposed MCSPSOSPEA outperforms other compatible rivals including conventional arrays by detecting radiated pattern with the highest directivity, lowest average SLL suppression, narrowest HPBW, and best predefined nulls mitigation, respectively.

In this research, the SPEA is the chosen Pareto optimum standard mechanism, which provides the hybrid MCSPSO algorithm to search further non-dominated solutions, and importantly without getting bias to any of the three objective functions involved. The research basically focuses on the preliminary analysis of MO optimization using various proposed MCS hybrid algorithms via global Pareto front for rectangular antenna array synthesis. Practically, despite generating the best performance results, the MCSPSOSPEA rectangular array design have two main drawbacks, which are the largest aperture size (inter-element distance), and highest power consumption (current feed amplitude). In the future, there will be objective functions included, which will offset/reduce these drawbacks while performing the Pareto MO optimization approach. Besides, this is a preliminary research, which executes the optimal results with a one run using 1000 iterations to ensure enough convergence for all the proposed MCS hybrid algorithms. Again, in the future, these proposed algorithms could be run many times and the average of the best results could be taken. Finally, there are several possibilities to verify the proposed MCS hybrid algorithms with other Pareto optimum methods, e.g. non-dominated sorting genetic algorithm (NSGA), vector evaluated genetic algorithm (VEGA), and niched Pareto genetic algorithm (NPGA) in a more complex antenna array geometry such as, concentric circular, cylindrical, spherical antenna with large number of asymmetric/non-uniform array elements under different signal processing windows, such as Tukey, Kaiser, Blackman, Lanczos, Hamming, and Poisson.

## Additional Information

**How to cite this article**: Abdul Rani, K. N. *et al*. Hybridization of Strength Pareto Multiobjective Optimization with Modified Cuckoo Search Algorithm for Rectangular Array. *Sci. Rep.* **7**, 46521; doi: 10.1038/srep46521 (2017).

**Publisher's note:** Springer Nature remains neutral with regard to jurisdictional claims in published maps and institutional affiliations.

## Figures and Tables

**Table 1:** Selected Optimal Pareto Fitness for SPEA-based Rectangular Arrays 2(*M* × *N*) = 20 × 20, Dolph–Chebyshev, maxIter = 1000).

Fitness	f_1_	f_2_	f_3_
SPEA	0.1524	0.0138	2.5550
MCSSPEA	0.1310	0.0135	2.7271
MCSHCSPEA	0.1292	0.0180	2.6517
MCSPSOSPEA	0.1066	0.0235	2.4824

**Table 2:** Optimal Symmetric Location for SPEA-based Rectangular Arrays 2(*M* × *N*) = 20 × 20, Dolph-Chebyshev, maxIter = 1000).

*Element*	1	2	3	4	5
*x*_*m*_ or *x*_*n*_ [*λ*/2]	±0.5000	±1.5000	±2.5000	±3.5000	±4.5000
SPEA	±0.5321	±1.5964	±2.6607	±3.7250	±4.7893
MCSSPEA	±0.4910	±1.4731	±2.4551	±3.4372	±4.4192
MCSHCSPEA	±0.5328	±1.5985	±2.6641	±3.7298	±4.7954
MCSPSOSPEA	±0.8026	±2.4078	±4.0130	±5.6182	±7.2234
***Element***	**6**	**7**	**8**	**9**	**10**
*x*_*m*_ or *x*_*n*_ [*λ*/2]	±5.5000	±6.5000	±7.5000	±8.5000	±9.5000
SPEA	±5.8535	±6.9178	±7.9821	±9.0464	±10.1107
MCSSPEA	±5.4013	±6.3834	±7.3654	±8.3475	±9.3295
MCSHCSPEA	±5.8610	±6.9267	±7.9923	±9.0580	±10.1236
MCSPSOSPEA	±8.8286	±10.4338	±12.0390	±13.6442	±15.2494

**Table 3:** Optimal Excitation Amplitude for SPEA-based Rectangular Arrays 2(*M* × *N*) = 20 × 20, Dolph-Chebyshev, maxIter = 1000).

*Element*	1	2	3	4	5
*I*_*m*_ or *I*_*n*_	1.0000	0.8771	1.2009	1.5497	1.9052
SPEA	0.9515	0.8486	1.2416	1.5357	1.9395
MCSSPEA	0.9951	0.8602	1.1706	1.5646	1.9606
MCSHCSPEA	1.0924	0.9338	1.2310	1.6770	1.9488
MCSPSOSPEA	1.6454	1.4067	1.8543	2.5261	2.9355
***Element***	**6**	**7**	**8**	**9**	**10**
*I*_*m*_ or *I*_*n*_	2.2465	2.5522	2.8022	2.9793	3.0712
SPEA	2.3027	2.6042	2.8458	3.0166	2.9654
MCSSPEA	2.3304	2.6700	2.8287	3.0153	3.1945
MCSHCSPEA	2.4240	2.7245	3.0340	3.2316	3.4101
MCSPSOSPEA	3.6514	4.1040	4.5702	4.8679	5.1367

**Table 4:** Optimal Excitation Phase for SPEA-based Rectangular Arrays 2(*M* × *N*) = 20 × 20, Dolph-Chebyshev, maxIter = 1000).

*Element*	1	2	3	4	5
*φ*_*m*_ or *φ*_*n*_	0°	163.0425°	22.8576°	164.4077°	113.8247°
SPEA	0°	173.5232°	24.3269°	174.9760°	121.1415°
MCSSPEA	0°	157.5868°	22.0928°	158.9062°	110.0158°
MCSHCSPEA	0°	173.7452°	24.3581°	175.2000°	121.2965°
MCSPSOSPEA	0°	180°	36.6911°	180°	180°
***Element***	**6**	**7**	**8**	**9**	**10**
*φ*_*m*_ or *φ*_*n*_	17.5573°	50.1297°	98.4387°	172.3512°	173.6799°
SPEA	18.6859°	53.3521°	104.7665°	180°	180°
MCSSPEA	16.9698°	48.4522°	95.1447°	166.5840°	167.8682°
MCSHCSPEA	18.7098°	53.4204°	104.9005°	180°	180°
MCSPSOSPEA	28.1830°	80.4683°	158.0140°	180°	180°

**Table 5:** Selected Optimal Pareto Fitness for SPEA-based Rectangular Arrays 2(*M* × *N*) = 20 × 20, Uniform, Null = [55°, 60°, 120°, 125°], maxIter = 1000).

Fitness	f_1_	f_2_	f_3_
SPEA	0.1454	0.0258	0.1277
MCSSPEA	0.1381	0.0222	0.1825
MCSHCSPEA	0.1379	0.0273	0.1825
MCSPSOSPEA	0.1292	0.0257	0.1280

**Table 6:** Optimal Symmetric Location for SPEA-based Rectangular Arrays 2(*M* × *N*) = 20 × 20, Uniform, Null = [55°, 60°, 120°, 125°], maxIter = 1000).

*Element*	1	2	3	4	5
*x*_*m*_ or *x*_*n*_ [*λ*/2]	±0.5000	±1.5000	±2.5000	±3.5000	±4.5000
SPEA	±0.5422	±1.6265	±2.7109	±3.7953	±4.8796
MCSSPEA	±0.5664	±1.6992	±2.8321	±3.9649	±5.0977
MCSHCSPEA	±0.6063	±1.8188	±3.0313	±4.2438	±5.4564
MCSPSOSPEA	±0.6578	±1.9735	±3.2892	±4.6049	±5.9206
***Element***	**6**	**7**	**8**	**9**	**10**
*x*_*m*_ or *x*_*n*_ [*λ*/2]	±5.5000	±6.5000	±7.5000	±8.5000	±9.5000
SPEA	±5.9640	±7.0484	±8.1327	±9.2171	±10.3014
MCSSPEA	±6.2305	±7.3633	±8.4962	±9.6290	±±10.7618
MCSHCSPEA	±6.6689	±7.8814	±9.0939	±10.3065	±11.5190
MCSPSOSPEA	±7.2363	±8.5520	±9.8677	±11.1834	±12.4991

**Table 7:** Optimal Excitation Amplitude for SPEA-based Rectangular Arrays 2(*M* × *N*) = 20 × 20, Uniform, Null = [55°, 60°, 120°, 125°], maxIter = 1000).

*Element*	1	2	3	4	5
*I*_*m*_ or *I*_*n*_	1.0000	1.0000	1.0000	1.0000	1.0000
SPEA	0.9524	1.0012	1.0740	0.9658	0.9869
MCSSPEA	0.9611	0.9952	1.1365	1.0450	1.1028
MCSHCSPEA	1.0287	1.0652	1.2165	1.1186	1.1804
MCSPSOSPEA	1.1162	1.1559	1.3200	1.2137	1.2808
***Element***	**6**	**7**	**8**	**9**	**10**
*I*_*m*_ or *I*_*n*_	1.0000	1.0000	1.0000	1.0000	1.0000
SPEA	1.0606	0.9731	0.9794	0.9840	0.9980
MCSSPEA	1.1177	1.1077	1.0975	1.0911	0.9911
MCSHCSPEA	1.1964	1.1857	1.1747	1.1678	1.0608
MCSPSOSPEA	1.2982	1.2866	1.2747	1.2672	1.1511

**Table 8:** Optimal Excitation Phase for SPEA-based Rectangular Arrays 2(*M* × *N*) = 20 × 20, Uniform, Null = [55°, 60°, 120°, 125°], maxIter = 1000).

*Element*	1	2	3	4	5
*φ*_*m*_ or *φ*_*n*_	0°	33.8087°	44.3390°	19.4383°	35.3010°
SPEA	0°	36.6609°	48.0796°	21.0782°	38.2791°
MCSSPEA	0°	38.2992°	50.2282°	22.0202°	39.9897°
MCSHCSPEA	0°	40.9939°	53.7622°	23.5695°	42.8034°
MCSPSOSPEA	0°	44.4820°	58.3368°	25.5750°	46.4454°
***Element***	**6**	**7**	**8**	**9**	**10**
*φ*_*m*_ or *φ*_*n*_	75.5787°	162.1825°	114.5353°	159.5137°	137.1483°
SPEA	81.9547°	175.8646°	124.1978°	172.9707°	148.7185°
MCSSPEA	85.6171°	180°	129.7480°	180°	155.3645°
MCSHCSPEA	91.6411°	180°	138.8770°	180°	166.2959°
MCSPSOSPEA	99.4387°	180°	150.6938°	180°	180°
